# Inhibition of cell-cycle progression by acute treatment with various degrees of hypoxia: modifications induced by low concentrations of misonidazole present during hypoxia.

**DOI:** 10.1038/bjc.1983.271

**Published:** 1983-12

**Authors:** E. O. Pettersen, T. Lindmo

## Abstract

The effect on cell-cycle progression in various phases of the cell cycle caused by an acute exposure to hypoxia in absence and presence of misonidazole (MISO) was investigated. Exponentially growing and synchronized cells of the human line NHIK 3025 were exposed to different degrees of hypoxia for a short period (1.5 or 3 h). The cell-cycle progression was studied both during and after hypoxia by flow-cytometric recording of DNA-histograms from treated and untreated cells. The rate of cell-cycle progression was reduced during hypoxia only if the O2-concentration was below 1000 ppm. The inhibition was phase specific with a strong effect in S (reduced DNA-synthesis), and a specific cell-cycle inhibition in late G1, probably at the G1/S-border. For cells inhibited (or arrested for extreme hypoxia) at the G1/S-border, the cell-cycle progression changed back to normal shortly after aerobic conditions were re-established. For cells rendered hypoxic and inhibited during S, hypoxia exerted a lasting effect expressed by a low cell-cycle progression rate even after aerobic conditions were re-established. This effect was strongly dependent on both the degree and the duration of the hypoxic treatment. The presence of a low concentration of MISO (0.05 mM) during hypoxia did not affect the cell-cycle progression during hypoxia at any O2-concentration. For cells rendered hypoxic during S, however, MISO (0.05 mM) counteracted the lasting effect of hypoxia for all concentrations of O2 where this lasting effect was observed.


*Br. J. Cancer* (1983), **48**, 809–817

### Inhibition of cell-cycle progression by acute treatment with various degrees of hypoxia: Modifications induced by low concentrations of misonidazole present during hypoxia

E.O. Pettersen[1] & T. Lindmo[2]

*Departments of [1]Tissue Culture and [2]Biophysics, Norsk Hydro's Institute for Cancer Research, The Norwegian Radium Hospital, Montebello, Oslo 3, Norway.*

**Summary** The effect on cell-cycle progression in various phases of the cell cycle caused by an acute exposure to hypoxia in absence and presence of misonidazole (MISO) was investigated. Exponentially growing and synchronized cells of the human line NHIK 3025 were exposed to different degrees of hypoxia for a short period (1.5 or 3 h). The cell-cycle progression was studied both during and after hypoxia by flow-cytometric recording of DNA-histograms from treated and untreated cells. The rate of cell-cycle progression was reduced during hypoxia only if the $O_2$-concentration was below 1000 ppm. The inhibition was phase specific with a strong effect in S (reduced DNA-synthesis), and a specific cell-cycle inhibition in late G1, probably at the G1/S-border. For cells inhibited (or arrested for extreme hypoxia) at the G1/S-border, the cell-cycle progression changed back to normal shortly after aerobic conditions were re-established. For cells rendered hypoxic and inhibited during S, hypoxia exerted a lasting effect expressed by a low cell-cycle progression rate even after aerobic conditions were re-established. This effect was strongly dependent on both the degree and the duration of the hypoxic treatment. The presence of a low concentration of MISO (0.05 mM) during hypoxia did not affect the cell-cycle progression during hypoxia at any $O_2$-concentration. For cells rendered hypoxic during S, however, MISO (0.05 mM) counteracted the lasting effect of hypoxia for all concentrations of $O_2$ where this lasting effect was observed.

Extensive research has aimed at eliminating the radioresistance of hypoxic tumour cells, for example by introducing chemicals which may specifically sensitize hypoxic cells (for overview see Adams *et al.*, 1978). Less effort has beeen made to map the biological behaviour of cells under hypoxic as compared to aerobic conditions. From studies on Chinese hamster cells it is known that the duration of G1 in particular, but also of S, is increased by hypoxia, whereas G2 and mitosis are hardly influenced (Bedford & Mitchell, 1974; Koch *et al.*, 1973; Born *et al.*, 1976). Little is known, however, about the variability of this effect between mammalian cells of different origin and about the mechanisms which cause this change in cell-cycle kinetics. Furthermore, little is known about the variation within G1 and S with respect to the sensitivity to hypoxia itself. This question may be of importance to radiotherapy due to great variation in radiosensitivity throughout the cell cycle. While cells in early G1 are among the most radioresistant in the cell cycle, cells at the G1/S border are among the most sensitive (Terasima & Tolmach, 1963; Hahn & Bagshaw, 1966; Sinclair, 1968; Pettersen *et al.*, 1977*a*).

Correspondence: E.O. Pettersen
Received 11 April 1983; accepted 15 August 1983.

In our previous paper (Pettersen & Lindmo, 1981) we showed that human NHIK 3025 cells that were rendered extremely hypoxic (<4 ppm $O_2$), accumulated at the G1/S border, while those in S stopped synthesizing DNA. Cells in G2 and mitosis continued into G1 during hypoxia without much delay. The cells arrested at the G1/S border tolerated hypoxia well (100% survival after 12 h of extreme hypoxia) while cells arrested in S were far more sensitive (40% survival after 3 h of extreme hypoxia).

For cells rendered extremely hypoxic for a short period of 3 h we found a division delay of about 3 h for G1-cells, but 15 h for S-cells. Thus, cells in S at the start of hypoxia have a slow cell-cycle progression also after reoxygenation. This lasting effect of hypoxia was, however, counteracted when small concentrations of hypoxic cell sensitizer (MISO) were present during hypoxia. The counteractive effect of MISO was observed for concentrations down to 0.01 mM, it was optimal at 0.05 mM and was seen only for cells which were in S during hypoxia. For concentrations of MISO above 0.5 mM during hypoxia we found a strong inactivating effect of the drug irrespective of cell-cycle phase, in line with earlier reports (Stratford & Adams, 1977; Hall & Roizin-Towle, 1975).

In the present experiments we changed the respiration rate of our NHIK 3025 cells by

© The Macmillan Press Ltd., 1983

exposing them to various degrees of hypoxia for a limited time period. According to Froese (1962) and Boag (1970) ascites tumour cells in suspension have full respiration for concentrations of $O_2$ above 1000 ppm. We therefore varied the oxygen concentration over a range from <4 to 1000 ppm. We studied cell-cycle inhibitory effects both in the absence and in presence of MISO and analysed specifically the inhibition induced during hypoxia at the G1/S-border and in S. In addition, the division delay of cells exposed to hypoxia for a short period in S was studied.

### Materials and methods

Human cells, NHIK 3025, established from a cervical carcinoma *in situ* (Nordbye & Oftebro, 1969; Oftebro & Nordbye, 1969) were cultivated as monolayers in medium E2a (Puck *et al.*, 1957), supplemented with 20% human serum (prepared in the laboratory) and 10% horse serum (Gibco, Scotland). The cells were routinely recultured 3 times weekly. There is no significant delay in growth rate after reculturing of the cells (Pettersen *et al.*, 1977*b*). As long as the cells are not allowed to grow to confluence the median cell-cycle time is 18 h, and under such growth conditions the cells meet the requirements set up by Anderson *et al.* (1967) for cells in balanced growth (Pettersen *et al.*, 1977*b*). The median durations of the various phases of the cell-cycle are G1 ~6.5 h, S ~8 h, G2 ~2.5 h and M ~1 h.

Populations with a high degree of synchrony were obtained by shake-off of mitotic cells from exponentially growing populations as described previously (Pettersen *et al.*, 1977*b*, Pettersen & Lindmo, 1981).

Usually the mitotic selection was repeated several times at 45 min intervals to provide enough synchronized cells for one experiment. To avoid unnecessary disturbance of the cells no attempt was made to accumulate cells from different selections. Thus, populations from different selections had different starting times. For each selection the yield of cells was $4–8 \times 10^5$ cells in 240 ml of medium. Immediately after the selection all the cells were seeded in 6–8 glass Petri dishes (Anumbra, Czechoslovakia) and placed in a $CO_2$ incubator operated at 5% $CO_2$ and 37°C.

The exposure to hypoxia (in absence and presence of MISO) has been described in detail in our earlier report (Pettersen & Lindmo, 1981). Briefly the glass Petri dishes containing 3 ml medium were placed without lids in stainless steel chambers and flushed with $N_2$ containing 3% $CO_2$ and various concentrations of $O_2$, using the set-up described earlier (Pettersen *et al.*, 1973; Løvhaug *et al.*, 1977). MISO was added as described (Pettersen & Lindmo, 1981) before the flushing was started. In order to prevent evaporation of medium from the dishes, the gas mixture was humidified before entering the chamber by passing through a sealed water bath having a separate temperature control operated at 37°C. After flushing, the medium in all dishes was replaced by 10 ml MISO-free, well-oxygenated medium. Both the mitotic selection and the flushing procedure took place in a walk-in incubator room at 37°C. Untreated control populations were kept in the $CO_2$ incubator all the time after mitotic selection.

Cell-cycle progression of synchronized cells was recorded by flow cytometric measurement of DNA histograms. At various times after mitotic selection, samples were trypsinized and stained with mithramycin (Mithracin, Pfizer Inc., USA) without previous fixation (Crissman & Tobey, 1974) as described earlier (Lindmo & Pettersen, 1979; Lindmo *et al.*, 1979). DNA-histograms were recorded on two different laboratory-built flow-cytometers (Lindmo & Steen, 1977; Pettersen *et al.*, in press). A pre-set number of cells was measured for each sample, and the histograms were analyzed as described earlier (Rønning *et al.*, 1981) to determine the fraction of cells in G1, S and G2+M (M for mitosis).

Inhibition at the G1/S border was studied by using asynchronous, exponentially growing cells. The cells were grown and rendered hypoxic as described for the synchronized cells. Samples were trypsinized and stained for flow-cytometric recording of DNA-histograms either immediately after or 3 h after the end of the hypoxic period (which also lasted for 3 h). The characteristic shape of the DNA-histograms 3 h after the end of hypoxia demonstrates, without further analysis, the degree of inhibition at the G1/S-border.

### Results

### *Inhibition at the G1/S border*

Figure 1 shows DNA-histograms of NHIK 3025 cells that were growing exponentially at the start of hypoxia and were fixed either immediately after the 3 h period of hypoxia or 3 h after the end of hypoxia. The data on <4 ppm $O_2$ are not included here since such data were shown earlier (Pettersen & Lindmo, 1981) and were similar to those at 21 ppm. Studying the histograms a, b and c in the upper row (21 ppm), one can see that at 3 h after hypoxia both histograms (b and c) have an extra peak in the early S whereas no such peak is present immediately after hypoxia (a). Nor is it present in

**Figure 1** DNA-histograms of exponentially growing NHIK 3025 cells, all treated with hypoxia for 3 h. The 5 histograms in the left hand column (a, d, g. j, m) represent cells trypsinized and stained immediately after hypoxia, while the two other columns represent cells trypsinized and stained 3 h after the end of hypoxia.

the untreated control which is fixed at the same times (m, n, o). The peak therefore represents cells that were accumulated and arrested at the G1/S border during hypoxia. During the period of 3 h after the end of hypoxia these cells have reached the stage in S where the extra peak is observed. A similar peak is also seen in populations treated with 86 or 197 ppm $O_2$, although in these cases the number of cells contained within the extra peak is lower (e and f; h and i). This indicates that in these cases the inhibition at the G1/S border is weaker than with 21 ppm $O_2$. In populations treated with 490 ppm $O_2$ no extra peak in S is seen 3 h after treatment. Two points can be made from Figure 1: Firstly, the inhibition of cell-cycle progression at the G1/S border is strong (possibly complete arrest) when the concentration of $O_2$ is $\leqslant 21$ ppm, but decreased with increasing $O_2$-concentration up to 490 ppm where the effect is absent. Secondly, MISO, when present during hypoxia (0.05 mM), does not influence the inhibition of cell-cycle progression at the G1/S border.

*Inhibition of DNA-synthesis*

Figure 2 shows DNA-histograms of synchronized cells. Histograms a, c, e and g demonstrate the normal cell-cycle progression of untreated cells during the period from 10–13 h after mitotic selection. At 10 h most of the cells have entered S, while some cells (~20%) still remain in G1. During the time period up to 13 h the fraction remaining in G1 decreases as the most slowly cycling cells enter S. During the same period the peak representing cells in S moved towards higher channel numbers, demonstrating that the cells synthesize DNA during this time period.

Histograms e–h represent cells treated with extreme hypoxia (4 ppm $O_2$) in absence or in presence of the indicated concentrations of MISO during the period from 10 to 13 h after mitotic selection. These cells were trypsinized and stained immediately after hypoxia to see whether DNA was synthesized during hypoxia. All 4 histograms e–h are quite similar to that at 10 h for the control, indicating that the amount of DNA has been unchanged during the period of extreme hypoxia. This also implies, as was shown in Figure 1, that cells in G1 were unable to enter S during this same period. Thus, there is no or very little DNA-synthesis under conditions of extreme hypoxia.

**Figure 2** DNA-histograms of synchronized NHIK 3025 cells. The panels a, c, e and g represent synchronized, untreated controls trypsinized and stained at the indicated times after mitotic selection. The cells represented in panels b, d, f and h were all trypsinized and stained at 13 h, immediately after a 3 h period of hypoxic treatment. The indicated concentrations of MISO were present only during hypoxia. The small peaks at about channel number 20 represent latex test particles that were mixed into the cell suspension after staining. The vertical lines at channel numbers 50 and 100 represent the amount of DNA in G1 and G2-cells respectively.

Furthermore, the presence of MISO (up to 0.4 mM) during hypoxia does not diminish this inhibition of DNA-synthesis.

Experiments like those shown in Figure 2 were also performed with higher concentrations of oxygen during hypoxia. The increase in DNA during the hypoxic period was in each case measured as the difference in mean channel number of histograms recorded immediately before and after hypoxia. The results (Figure 3) show that there is no accumulation of DNA with <4 or 21 ppm $O_2$ under the hypoxic conditions. With higher concentrations of $O_2$, however the rate of DNA synthesis increases with increasing $O_2$ and is 100% for 1000 ppm $O_2$. MISO, when present at a concentration of 0.05 mM during hypoxia, had no effect on the rate of DNA-synthesis for any concentration of oxygen.

**Figure 3** The increase in the mean amount of DNA per cell over a 3 h period from 10–13 h after mitotic selection as a function of the oxygen concentration during this period. Data are shown relative to untreated control cells. O: hypoxia only; ●, hypoxia + 0.05 mM MISO. The vertical bars represent s.e. of the mean from 2 or 3 experiments.

*Division delay after hypoxic treatment in S*

In our previous report (Pettersen & Lindmo, 1981) we showed that cells treated for 3 h with extreme hypoxia during S were severely inhibited in their cell-cycle progression after the period of hypoxia. We have presently measured this effect for various concentrations of oxygen during hypoxia. For this purpose we measure the parameter "division delay" which is the increase in cell-cycle as illustrated in Figure 4. The period of hypoxia started 10 h after mitotic selection and lasted for 1.5 or 3 h. From analysis of DNA-histograms recorded at various intervals after hypoxia the fraction of the initial cells which has not divided was calculated as described in Appendix 1. Initial cells denote the cells traversing the first interphase after mitotic selection. The median cell-cycle duration was taken as the time from 0.5 h after mitotic selection when 50% of the cells had divided for the first time until 50% of the cells had divided for the second time (Pettersen *et al.*, 1977*b*). The division delay is the difference between the median cell-cycle times for the treated and untreated (aerobic) cell populations. In Figure 4 this parameter is denoted "increase in cell-cycle" for the population treated from 10–13 h after mitotic selection with 21 ppm $O_2$ + 0.05 mM MISO.

In Figure 4 the slopes of the curves express the spread in cell-cycle times of the various populations. As illustrated by the data the spread in cell-cycle duration is greater in the treated than in the untreated cell populations and greater after 21 than after 197 ppm $O_2$.

Figure 5 shoes the division delay as a function of the concentration of oxygen during the hypoxia with 4 different types of treatment: 1.5 h hypoxia (triangles) or 3 h hypoxia (circles) in absence (open) or in presence (closed) or 0.05 mM MISO. After 3 h treatment the division delay is >13 h when the $O_2$-concentration is <4 or 21 ppm. From Figures 2 and 3 we know that at these low concentrations of $O_2$ there is no cell-cycle progression during hypoxia. Thus, of the 13 h division delay 3 h were accumulated during hypoxia, and 10 h accumulated after hypoxia. Compared to untreated cells the rate of cell-cycle progression is therefore reduced after hypoxia (a lasting effect of hypoxia) when the $O_2$-concentration during hypoxia is very low (<200 ppm and <20 ppm for 3 h and 1.5 h hypoxia, respectively). These findings are summarized in Figure 6.

MISO (0.05 mM), when present during hypoxia, only leads to a reduction in the lasting effect of hypoxia, but does not relieve the cells of cell-cycle inhibition during hypoxia.

### Discussion

*Effects of hypoxia alone*

The present results are in accordance with the findings of Bedford & Mitchell (1974) that cells grown under various degrees of hypoxia experience a prolongation of mainly G1 and S. However, from

**Figure 4** The fraction of the initial cells which have not divided as a function of the time after mitotic selection, as calculated from DNA-histograms. Data from an untreated control is shown together with 4 populations treated with hypoxia from 10 to 13 h after selection in absence or presence of 0.05 mM MISO. This figure demonstrates the measurement of the parameter called "division delay" (see **Figure 5**).

**Figure 5** The division delay for NHIK 3025 cells exposed to hypoxia in S as a function of the concentration of $O_2$. Two different periods of hypoxia was used: 1.5 h (10–11.5 h after mitotic selection—▲, △) and 3 h (10–13 h after mitotic selection—●, ○). The experimental points represent the mean of results from 2 or 3 experiments. S.e. are represented by vertical bars.

**Figure 6** Summary of the effects on cell-cycle progression of NHIK 3025 cells caused by acute hypoxia for 1.5 or 3 h in S. The vertical arrows indicate the concentration limits between which the indicated effects appear.

the present study it is possible to make more detailed statements about the effect within these two phases.

*Inhibition at the G1/S border* During hypoxia the cells are specifically inhibited at, or close to, the G1/S-border. From Figure 1 this effect is apparent for oxygen tensions corresponding to 197 ppm and lower, but not 490 ppm. In the report of Bedford & Mitchell (1974) it was shown for Chinese hamster CHO cells that G1 was also prolonged more than S for an $O_2$-concentration of 690 ppm. An explanation of this discrepancy may be that Bedford & Mitchell measured phase durations of cells which had been grown under hypoxic conditions for >36 h, while in the present study hypoxia lasted for $\not>$3 h. The inhibition at the G1/S border could be time-dependent, or, if the inhibition is weak for such high concentrations of $O_2$ as 490 ppm, 3 h could be too short a time to accumulate a detectable number of cells at the G1/S border.

However, the important observation in Figure 1 is that the degree of inhibition decreases with increasing $O_2$-concentration up to about 500 (or 1000) ppm. This coincides with the concentration limit above which the cellular respiration was normal in Ehrlich Ascites tumour cells (Froese, 1962; Boag, 1970). Thus, a fair assumption is that the inhibition at the G1/S border is a direct consequence of a reduction in cellular respiration.

*Inhibition in S* For cells rendered hypoxic while in S there are two limit concentrations of $O_2$ under our experimental conditions (see vertical arrows in Figure 6). The upper limit is 1000 ppm whether hypoxia lasts for 1.5 or 3 h. When the $O_2$-concentration is higher, the cell-cycle progression is not affected. For $O_2$-concentrations <1000 ppm the rate of cell-cycle progression is reduced at least during hypoxia. Since this limit coincides with that for which the cellular respiration is reduced (Froese, 1962; Boag, 1970) it is tempting to assume that the inhibition during S (as that at the G1/S-border) is a consequence of the reduction in cellular respiration. This is in accordance with the finding of Olivotto & Paoletti (1981) that recruitment of Yoshida ascites hepatoma cells into S strictly depends on the activity of the respiratory chain.

The lower limit depends on the duration of the period of hypoxia and is 20 ppm and 200 ppm for 1.5 h and 3 h hypoxia respectively (see vertical arrows in Figure 6). Above this limit (but <1000 ppm) the rate of cell-cycle progression is reduced during the period of hypoxia, but changes immediately back to normal when normal aerobic conditions are re-established. Below this limit, however, the rate of cell-cycle progression is below normal even after normal aerobic conditions are re-established. This is the effect we denote the lasting effect of hypoxia.

There are several possible reasons for the lasting effect of hypoxia on cells in S. For example, molecular damage could accumulate due to a reduced repair capacity during hypoxia. It is also possible that a reduced molecular synthesis during hypoxia might entail a lack of substances needed for growth. Hypoxia could even induce production of growth-inhibiting waste products. In all cases one would expect, as we have found (Figure 5), that the lasting effect of hypoxia would increase with both increasing duration and increasing degree of hypoxia. Thus, one cannot from the present data conclude which cellular or molecular mechanism secondary to reduced respiration produce the lasting effect of hypoxia for cells rendered hypoxic in S. We can, however, state that the effect is not seen for cells rendered hypoxic in G1. In our previous study (Pettersen & Lindmo, 1981) we exposed exponentially growing cells to extreme hypoxia for up to 12 h. The cells that were arrested at the G1/S-border during this time entered S-phase with a normal rate of cell-cycle progression after normal aerobic conditions were re-established.

As reported recently by Brock *et al.* (1982), gradient separated hypoxic tumour cells from a methylcholanthrene-induced murine fibrosarcoma, reoxygenated by plating in culture, immediately began to synthesize DNA at rates similar to well-oxygenated cells. From our present findings one would have expected a reduced DNA-synthesis in the fraction of the cells that were in S at the time of plating, if the concentration of oxygen in the hypoxic regions of the tumour was below ~100 ppm. However, the fraction of S-cells in the separated hypoxic cell populations was only 25% and may have been too low to give a significant reduction in over-all [$^3$H]dT-incorporation. It is also a possibility that the oxygen concentration in the hypoxic regions of the fibrosarcoma tumour varied and in many areas was above 100 ppm.

### *Effects of hypoxia in presence of 0.05 mM MISO*

As demonstrated in Figure 5 the counteractive effect of MISO on the lasting effect of hypoxia in S appears for all concentrations of $O_2$ where this effect of hypoxia is seen. The mechanism by which MISO exerts this counteraction is still obscure, as is the molecular mechanism by which the lasting effect of hypoxia is induced. However, whether the lasting effect of hypoxia is caused by molecular damage, increase in waste products or reduced synthesis of products needed for growth, MISO most likely affects these processes in a rather direct manner, and not by mimicking the respiratory effect of $O_2$ as we suggested in our former paper (Pettersen & Lindmo, 1981). If that had been the case, we would have expected MISO to exert a counteractive effect during hypoxia. No such effect was seen in any cell-cycle phase.

The authors gratefully acknowledge the technical assistance of Charlotte Borka and Ursula Prehn Hansen, and of Kåre Fundingsrud who skilfully operated the flow cytometer. This work was supported by the Norwegian Cancer Society—Landsforeningen mot Kreft.

### Appendix 1

### *The fraction of initial cells which have not divided*

In our previous paper (Pettersen *et al.*, 1977) we presented a formula giving the cell number as a function of the mitotic index (the fraction of cells in mitosis) in a population of selected mitoses where the cells start to divide immediately after selection:

$$C = \frac{2\,C_0}{1+m} \qquad (1)$$

$C$ is the cell number, $C_0$ is the initial cell number (the number of selected mitoses) and $m$ is the mitotic index. In such a population the fraction of cells which have not yet divided is $m$, and the number of cells which have not yet divided is $m \cdot C$. Since we want to calculate the fraction of the *initial* cells which have not yet divided ($f$) we get:

$$f = \frac{mC}{C_0} \qquad (2)$$

and when inserted for $C$:

$$f = \frac{2m}{1+m} \qquad (3)$$

Although formula (3) describes the first period of division after mitotic selection, it is equally well applicable for the next one where initial cells will denote cells traversing the first interphase after mitotic selection. However, in that case the undivided cells are not exclusively in mitosis but are distributed among late S, G2 and mitosis (because of the natural decay of synchrony with time). Therefore $m$ must in that case be taken to mean the fraction of cells in those stages of the cell cycle. This fraction is determined from the flow-cytometry data, and is used in formula (3) to determine the fraction of the initial cells which have not yet divided.

### References

ADAMS, G.E., FOWLER, J.F. & WARDMAN, P. (Eds.). (1978). Hypoxic cell sensitizers in radiobiology and radiotherapy. *Br. J. Cancer*, **37**, Suppl. III.

ANDERSON, E.C., PETERSON, D.F. & TOBEY, R.A. (1967). Biochemical balance and synchronized cell cultures. *Nature*, **215**, 1083.

BEDFORD, J.S. & MITCHELL, J.B. (1974). The effect of hypoxia on the growth and radiation response of mammalian cells in culture. *Br. J. Radiol.*, **47**, 687.

BOAG, J.W. (1970). Cell respiration as a function of oxygen tension. *Int. J. Radiat. Biol.*, **18**, 475.

BORN, R., HUG, O. & TROTT, K.R. (1976). The effect of prolonged hypoxia on growth and viability of Chinese hamster cells. *Int. J. Radiat. Oncol. Biol. Phys.*, **1**, 687.

BROCK, W.A., SWARTZENDRUBER, D.E. & GRDINA, D.J. (1982). Kinetic heterogeneity in density-separated murine fibrosarcoma subpopulations. *Cancer Res.*, **42**, 4999.

CRISSMAN, H.A. & TOBEY, R.A. (1974). Cell-cycle analysis in 20 minutes. *Science*, **184**. 1297.

FROESE, G. (1962). The respiration of ascites tumour cells at low oxygen concentrations. *Biochim. Biophys. Acta*, **57**, 509.

HAHN, G.M. & BAGSHAW, M.A. (1966). Serum concentration: Effects on cycle and X-ray sensitivity of mammalian cells. *Science*, **151**, 459.

HALL, E.J. & ROIZIN-TOWLE, L. (1975). Hypoxic sensitizers: Radio-biological studies at the cellular level. *Radiology*, **117**, 453.

KOCH, C.J., KRUUV, J., FREY, H.E. & SNYDER, R.A. (1973). Plateau phase in growth induced by hypoxia. *Int. J. Radiat. Biol.*, **23**, 67.

LINDMO, T. & STEEN, H.B. (1977). Flow cytometric measurement of the polarization of fluorescence from intracellular fluorescein in mammalian cells. *Biophys. J.*, **18**, 173.

LINDMO, T., PETTERSEN, E.O. & WIBE, E. (1979). Cell-cycle inhibition by misonidazole of human cells cultivated *in vitro* under aerobic conditions. *Br. J. Cancer*, **40**, 755.

LINDMO, T. & PETTERSEN, E.O. (1979). Delay of cell cycle progression after X-irradiation of synchronized populations of human cells (NHIK 3025) in culture. *Cell Tissue Kinet.*, **12**, 43.

LØVHAUG, D., WIBE, E., OFTEBRO, R., PETTERSEN, E.O. & BRUSTAD, T. (1977). Recovery from X-ray induced damage in human cells grown in culture. *Neoplasma*, **24**, 513.

NORDBYE, K. & OFTEBRO, R. (1969). Establishment of four new cell strains from human uterine cervix. I. *Exp. Cell Res.*, **58**, 458.

OLIVOTTO, M. & PAOLETTI, F. (1981). The role of respiration in tumour cell transition from the noncycling to the cycling state. *J. Cell. Physiol.*, **107**, 243.

OFTEBRO, R. & NORDBYE, K. (1969). Establishment of four new cell strains from human uterine cervix. II. *Exp. Cell Res.*, **58**, 459.

PETTERSEN, E.O., OFTEBRO, R. & BRUSTAD, T. (1973). X-ray inactivation of human cells in tissue culture under aerobic and extremely hypoxic conditions in the presence and absence of TMPN. *Int. J. Radiat. Biol.*, **24**, 285.

PETTERSEN, E.O., CHRISTENSEN, T., BAKKE, O. & OFTEBRO, T. (1977*a*). A change in the oxygen effect throughout the cell-cycle of human cells of the line NHIK 3025 cultivated *in vitro*. *Int. J. Radiat. Biol.*, **31**, 171.

PETTERSEN, E.O., BAKKE, O., LINDMO, T. & OFTEBRO, R. (1977*b*). Cell cycle characteristics of synchronized and asynchronous populations of human cells and effect of cooling of mitotic cells. *Cell Tissue Kinet.*, **10**, 511.

PETTERSEN, E.O. & LINDMO, T. (1981). Low concentrations of misonidazole counteract effects of extreme hypoxia on cells in S. *Br. J. Cancer*, **43**, 355.

PETTERSEN, E.O., NOME, O., RØNNING, Ø.W. & OFTEBRO, R. (1983). Effects of benzaldehyde on survival and cell-cycle kinetics of human cells cultivated *in vitro*. *Eur. J. Cancer Clin. Oncol.*, **19**, 507.

PUCK, T.T., CIECIURA, S.J. & FISHER, H.W. (1957). Clonal growth *in vitro* of human cells with fibroblastic morphology. *J. Exp. Med.*, **106**, 145.

RØNNING, Ø.W., LINDMO, T., PETTERSEN, E.O. & SEGLEN, P.O. (1981). Kinetics of entry into S-phase and into the G1-phase of the subsequent cell cycle for synchronized NHIK 3025 cells. *Acta Pathol. Microbiol. Scand., Sect. A*, (Suppl.) **274**, 350.

SINCLAIR, W. (1968). Cyclic X-ray responses in mammalian cells *in vitro*. *Radiat. Res.*, **33**, 620.

STRATFORD, L.J. & ADAMS, G.E. (1977). Effect of hyperthermia on differential cytotoxicity of a hypoxic cell radiosensitizer, Ro-07-0582, on mammalian cells *in vitro*. *Br. J. Cancer*, **35**, 307.

TERASIMA, T. & TOLMACH, L.J. (1963). Variations in several responses of HeLa cells to X-irradiation during the division cycle. *Biophys. J.*, **3**, 11.


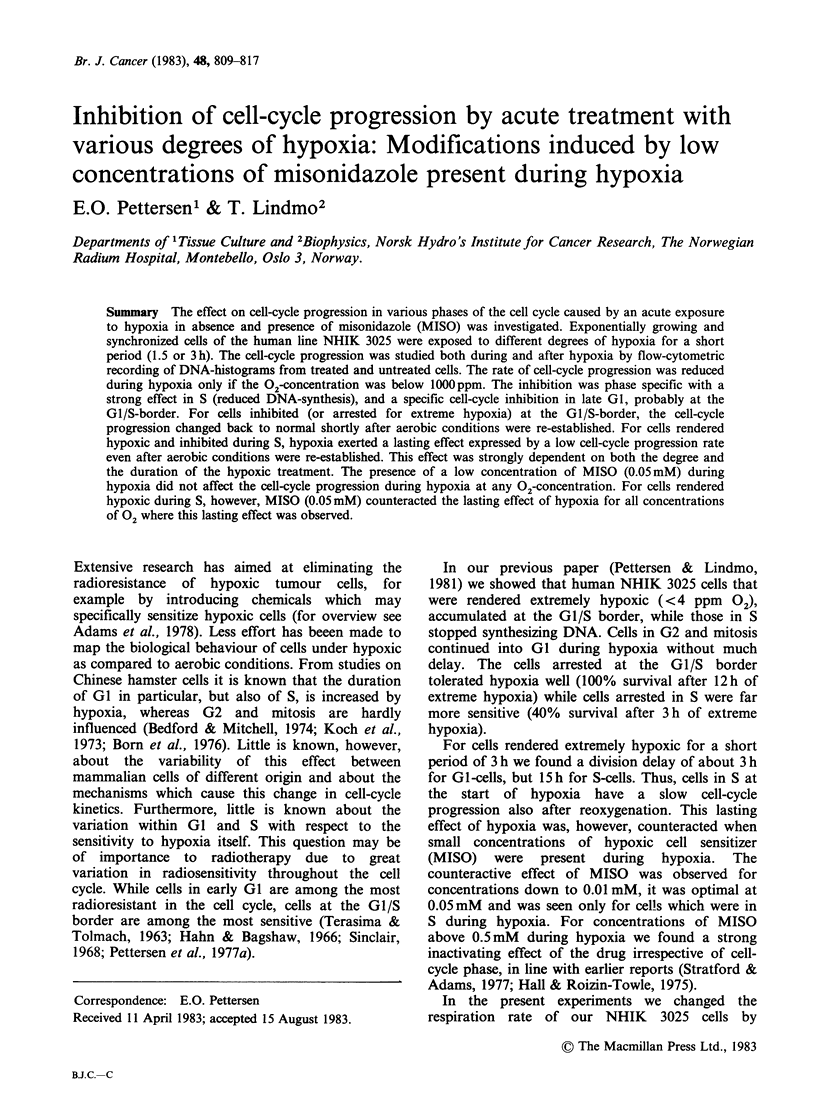

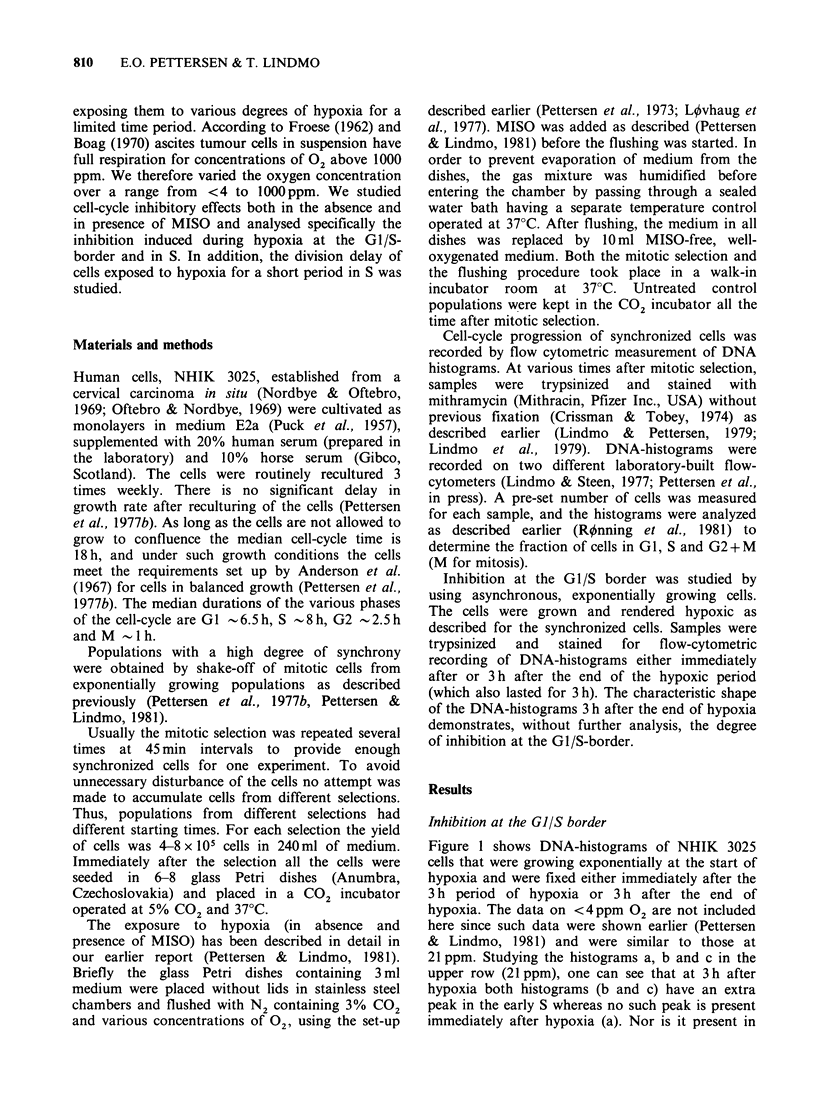

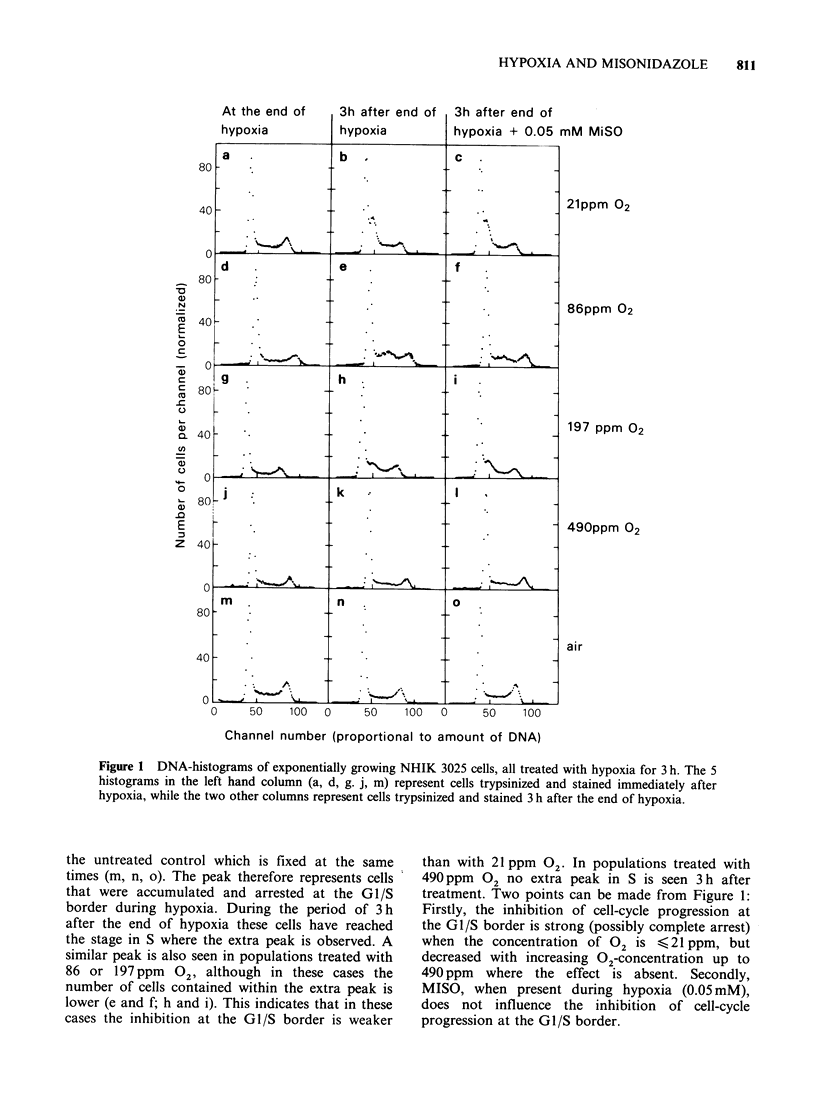

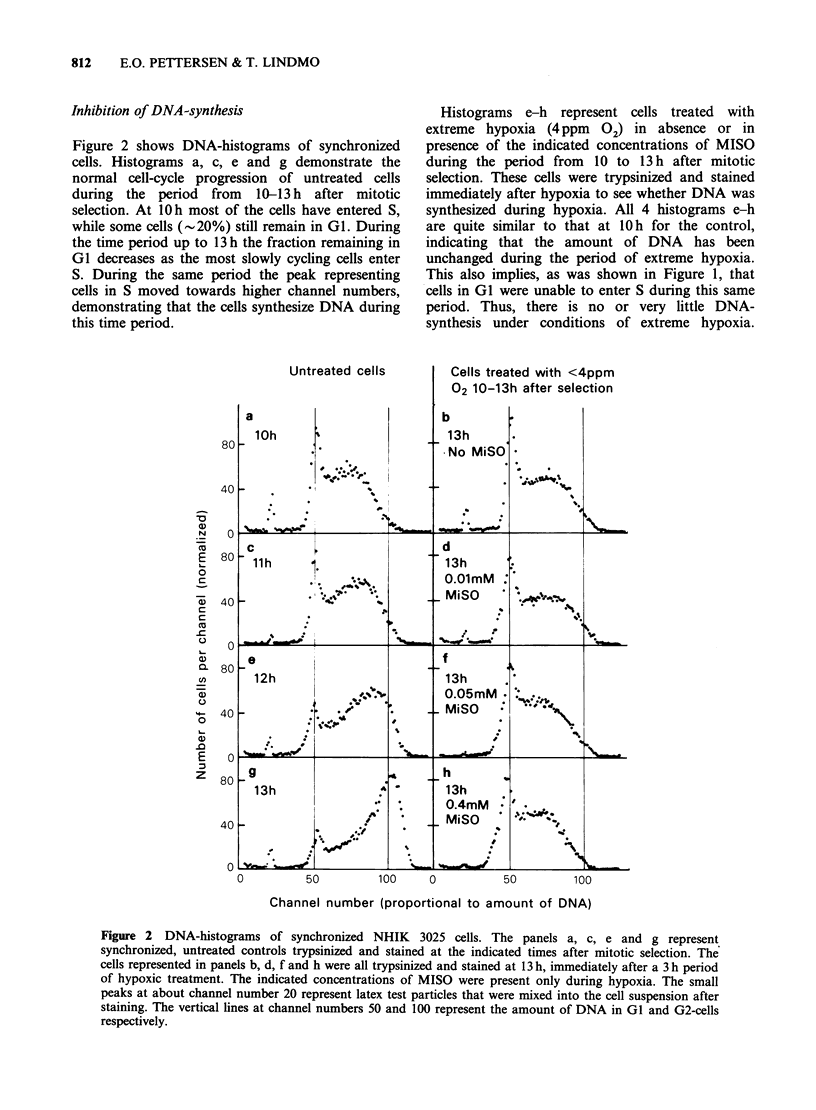

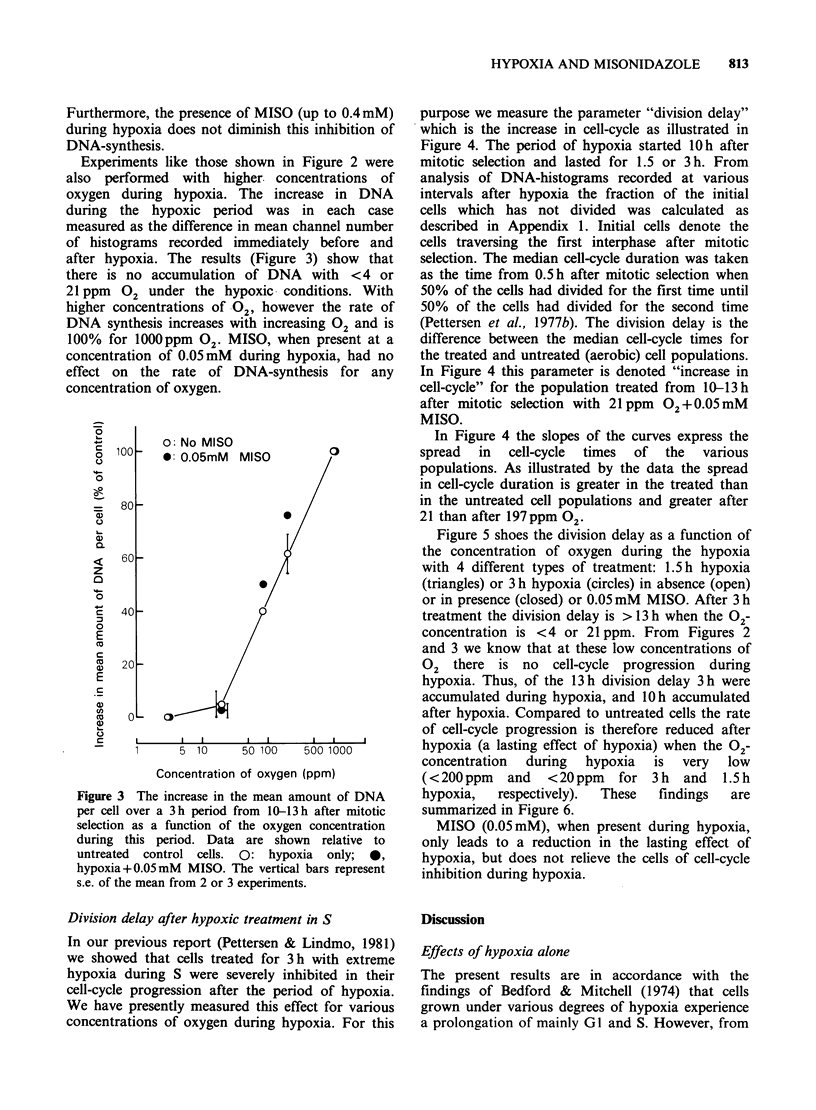

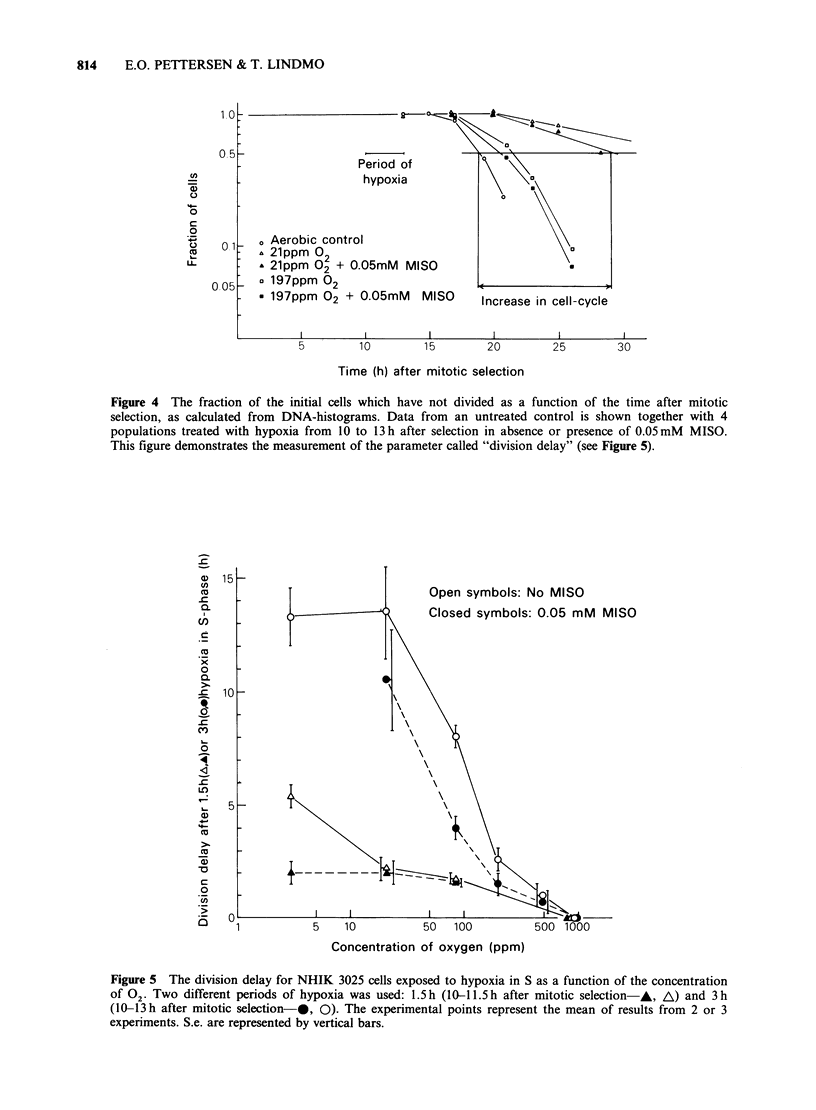

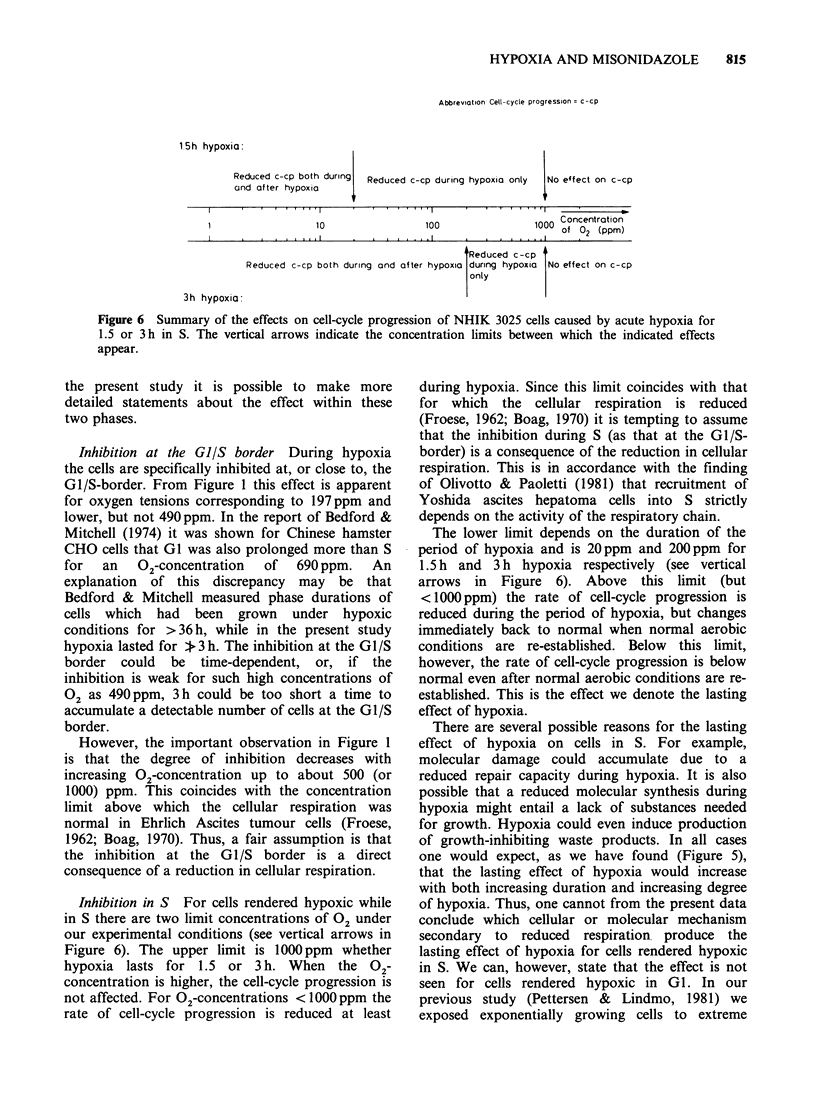

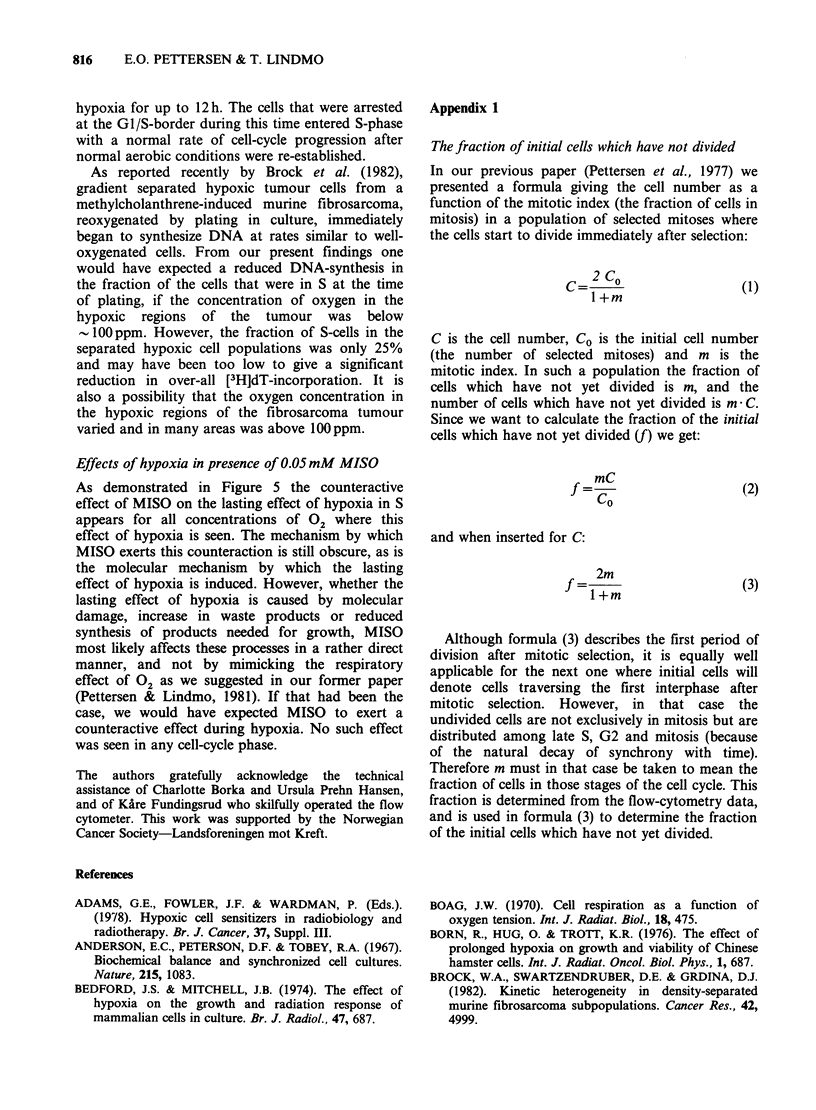

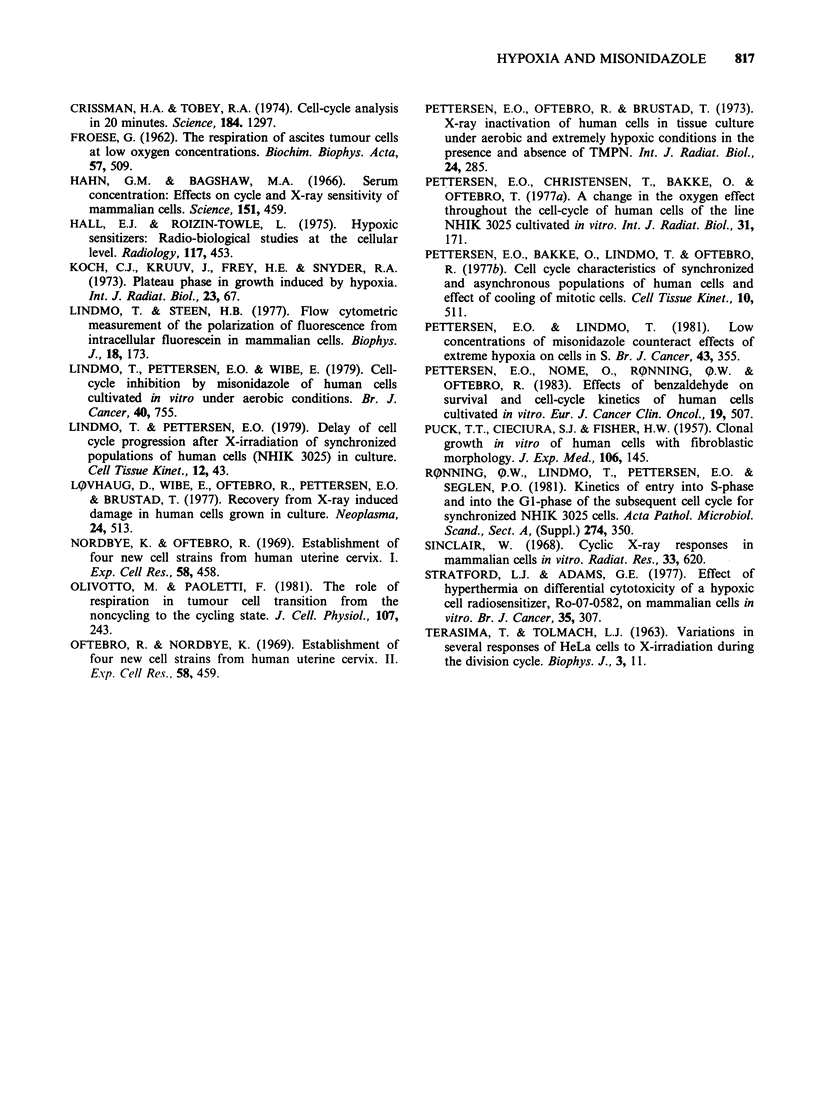


## References

[OCR_00909] Anderson E. C., Petersen D. F., Tobey R. A. (1967). Biochemical balance and syncronized cell cultures.. Nature.

[OCR_00914] Bedford J. S., Mitchell J. B. (1974). The effect of hypoxia on the growth and radiation response of mammalian cells in culture.. Br J Radiol.

[OCR_00919] Boag J. W. (1970). Cell respiration as a function of oxygen tension.. Int J Radiat Biol Relat Stud Phys Chem Med.

[OCR_00923] Born R., Hug O., Trott K. R. (1976). The effect of prolonged hypoxia on growth and viability of Chinese hamster cells.. Int J Radiat Oncol Biol Phys.

[OCR_00928] Brock W. A., Swartzendruber D. E., Grdina D. J. (1982). Kinetic heterogeneity in density-separated murine fibrosarcoma subpopulations.. Cancer Res.

[OCR_00936] Crissman H. A., Tobey R. A. (1974). Cell-cycle analysis in 20 minutes.. Science.

[OCR_00940] FROESE G. (1962). The respiration of ascites tumour cells at low oxygen concentrations.. Biochim Biophys Acta.

[OCR_00945] Hahn G. M., Bagshaw M. A. (1966). Serum concentration: effects on cycle and x-ray sensitivity of mammalian cells.. Science.

[OCR_00950] Hall E. J., Roizin-Towle L. (1975). Hypoxic sensitizers: radiobiological studies at the cellular level.. Radiology.

[OCR_00955] Koch C. J., Kruuv J., Frey H. E., Snyder R. A. (1973). Plateau phase in growth induced by hypoxia.. Int J Radiat Biol Relat Stud Phys Chem Med.

[OCR_00972] Lindmo T., Pettersen E. O. (1979). Delay of cell cycle progression after X-irradiation of synchronized populations of human cells (NHIK 3025) in culture.. Cell Tissue Kinet.

[OCR_00966] Lindmo T., Pettersen E. O., Wibe E. (1979). Cell-cycle inhibition by misonidazole of human cells cultivated in vitro under aerobic conditions.. Br J Cancer.

[OCR_00960] Lindmo T., Steen H. B. (1977). Flow cytometric measurement of the polarization of fluorescence from intracellular fluorescein in mammalian cells.. Biophys J.

[OCR_00978] Lovhaug D., Wibe E., Oftebro R., Pettersen E. O., Brustad T. (1977). Recovery from x-ray induced damage in human cells grown in culture.. Neoplasma.

[OCR_00989] Olivotto M., Paoletti F. (1981). The role of respiration in tumor cell transition from the noncycling to the cycling state.. J Cell Physiol.

[OCR_01031] PUCK T. T., CIECIURA S. J., FISHER H. W. (1957). Clonal growth in vitro of human cells with fibroblastic morphology; comparison of growth and genetic characteristics of single epithelioid and fibroblast-like cells from a variety of human organs.. J Exp Med.

[OCR_01014] Pettersen E. O., Bakke O., Lindmo T., Oftebro R. (1977). Cell cycle characteristics of synchronized and asynchronous populations of human cells and effect of cooling of selected mitotic cells.. Cell Tissue Kinet.

[OCR_01007] Pettersen E. O., Christensen T., Bakke O., Oftebro R. (1977). A change in the oxygen effect throughout the cell-cycle of human cells of the line NHIK 3025 cultivated in vitro.. Int J Radiat Biol Relat Stud Phys Chem Med.

[OCR_01021] Pettersen E. O., Lindmo T. (1981). Low concentrations of misonidazole counteract effects of extreme hypoxia on cells in S.. Br J Cancer.

[OCR_01028] Pettersen E. O., Nome O., Rønning O. W., Oftebro R. (1983). Effects of benzaldehyde on survival and cell-cycle kinetics of human cells cultivated in vitro.. Eur J Cancer Clin Oncol.

[OCR_01000] Pettersen E. O., Oftebro R., Brustad T. (1973). X-ray inactivation of human cells in tissue culture under aerobic and extremely hypoxic conditions in the presence and absence of TMPN.. Int J Radiat Biol Relat Stud Phys Chem Med.

[OCR_01043] Sinclair W. K. (1968). Cyclic x-ray responses in mammalian cells in vitro.. Radiat Res.

[OCR_01047] Stratford I. J., Adams G. E. (1977). Effect of hyperthermia on differential cytotoxicity of a hypoxic cell radiosensitizer, Ro-07-0582, on mammalian cells in vitro.. Br J Cancer.

[OCR_01053] TERASIMA T., TOLMACH L. J. (1963). Variations in several responses of HeLa cells to x-irradiation during the division cycle.. Biophys J.

